# Characteristics of ataxic gait in familial dysautonomia patients

**DOI:** 10.1371/journal.pone.0196599

**Published:** 2018-04-26

**Authors:** Sigal Portnoy, Channa Maayan, Jeanna Tsenter, Yonah Ofran, Vladimir Goldman, Nurit Hiller, Naama Karniel, Isabella Schwartz

**Affiliations:** 1 Department of Physical Medicine and Rehabilitation, Hadassah Medical Center, Hebrew University Hadassah medical school, Jerusalem, Israel; 2 Department of Occupational Therapy, Sackler Faculty of Medicine, Tel Aviv University, Tel Aviv, Israel; 3 Familial Dysautonomia Center, Pediatric department, Hadassah Medical Center, Hebrew University Hadassah medical school, Jerusalem, Israel; 4 Department of Orthopedic Surgery, Hadassah Medical Center, Hebrew University Hadassah medical school, Jerusalem, Israel; 5 Department of Radiology, Hadassah Medical Center, Hebrew University Hadassah medical school, Jerusalem, Israel; University of Illinois at Urbana-Champaign, UNITED STATES

## Abstract

**Introduction and objectives:**

Progressive ataxic gait is a common symptom in individuals with Familial Dysautonomia (FD). At least 50% of adults with FD require assistance with walking. Our aims were to describe the medical condition of individuals with FD (ii) compare their gait characteristics to healthy individuals, and (iii) assess correlations between gait measures, presence of unstable gait pattern and frequency of falls.

**Methods:**

Twelve subjects with FD (7 males, age 25.3±10.6 years) and 16 healthy participants (6 males, age 35.9±11.9 years) were recruited. Gait kinematics, gait symmetry, dynamic muscle activity, and foot deep vibration sensation were recorded.

**Results:**

Ataxic gait degrees were: severe (6 out of 12), moderate (4 out of 12) and low (2 out of 12). The number of falls correlated with base width asymmetry. Crouch gait was noted in 3 out of 12 of the subjects.

**Conclusions:**

In-depth quantitative gait analysis of individuals with FD revealed ataxic gait. The ataxic pattern might be a result of combined neurological deficiencies and osseous deformities. Increasing the base of support of patients with FD might increase the symmetry of the base width during gait and decrease the number of falls. Additionally, perturbation treatment and dynamic balance exercises may be recommended in order to improve compensatory strategies. Future investigation of this population should include quantification of osseous rotations of the lower limb in order to fully understand its effect on their gait pattern and falls.

## 1. Introduction

Familial Dysautonomia (FD), or Riley-Day syndrome, is a rare [1], autosomal recessive Hereditary Sensory and Autonomic Neuropathy type III (HSAN III), mainly found in individuals of Jewish descent. This disorder affects autonomic, sensory, and motor functions. Mutations in the IKBKAP gene located in chromosome 9q31-q33 were first discovered in 2001 [2–4]. The defect leads to abnormal development and progressive degeneration of the autonomic nervous systems which affects the function of most body systems. Common symptoms include aspirations and chronic lung disease, nasopharyngeal uncoordination and gastroesophageal reflux, variability of blood pressure, spinal curvature, hypotonia (especially in young patients), and osteoporosis. Additionally, progressive ataxic gait, i.e. uncoordinated and unbalanced gait [5–8] is a common symptom of FD. The ataxic gait characteristics in patients with FD were described as adaptation of a wide stance, arm lifting, and deviating into walls while taking unsteady turns [9]. In a recent review concerning gait ataxia [10], its symptoms have been described as increased step width, variable foot placement, irregular foot trajectories, unstable walking path with extremely high movement variability, and a high risk of falling. Consequently, at least 50% of adult individuals with FD require assistance with walking [6,7]. Several hypotheses regarding the gait abnormalities in individuals with FD have been suggested. There is little evidence of gross atrophy of the cerebellar vermis in patients with HSAN III that may cause gait ataxia [7]. An early hypothesis relies on histopathological findings that show a significant reduction in the number of neurons in the superior and the inferior divisions of the vestibular nerve that may account for gait imbalance and falls [11]. Alternatively, a more recent hypothesis suggests that the absence of muscle spindle afferents functionality and decrease in proprioceptive accuracy compromise the sensorimotor control of the gait and is therefore the main source of ataxia [7,12]. Unfortunately, individuals with FD may suffer a progressive loss of sight, which may further intensify their motor difficulties since, in the absence of proprioceptive feedback, they depend on visual feedback [9]. There is no validated quantitative method for assessing the level of severity of ataxic gait. A suggested calculation of the ratio of the Standard Deviation (SD) of foot placement: (SD of step length + SD of step width + SD of step height)/3, was calculated for tandem gait alone [13].

Several common symptoms of FD, e.g. the unsteady gait, cerebellar ataxia, impaired vision, scoliosis, kyphoscoliosis and drops in blood pressure, may be the source of increased incidence of falls [14]. The mean prevalence of fractures per FD patient is 1.5 [15], while in healthy population above the age of 50 years, the expected prevalence of fracture is 70% [16]. Furthermore, many patients with FD have osteoporosis, which may partially explain their increased risk of fractures following a fall. Since every fracture in this population may decrease dynamic stability while walking, it is important to prevent factors that may cause falls. Therefore, a comprehensive, patient-specific assessment of gait characteristics is crucial. Another factor that may contribute to the risk of falls is crouch gait, defined as excessive ankle dorsiflexion with knee and hip flexion during the stance phase [17]. However, to the best of our knowledge, crouch gait has yet to be documented in patients with FD.

Current technologies of motion capture and dynamic electromyography (EMG) allow precise quantification of gait characteristics and have been frequently utilized to study gait of individuals with different conditions, such as Cerebral Palsy [18,19], post-polio syndrome [20], and multiple sclerosis [21]. The acquired measurements were shown to provide insight into various pivotal predictors of functionality (predicted by gait velocity), risk of falls (predicted by gait symmetry), and fatigue (predicted by muscle activity duration).

In this study, we aimed to (i) describe the medical condition of individuals with FD (ii) compare their gait characteristics to healthy individuals, and (iii) assess correlations between gait measures, presence of unstable gait pattern and frequency of falls.

## 2. Methods

A cross-sectional study performed at a rehabilitation center.

### 2.1 Participants

Twelve individuals diagnosed with FD participated in this study. The subjects were recruited at the Familial Dysautonomia Center at the Hadassah Medical Center, Mount Scopus, Jerusalem, when arriving at for a routine examination (CM). Diagnosis of FD was based on their clinical symptoms, physical signs, skin Histamine test, and by the FD genetic test. Inclusion criteria were ability to walk without assistance of a caretaker, with or without walking aids. Exclusion criteria were cognitive disorders to assure that the participants could understand and sign the informed consent. Individuals with a gait abnormality that is not related to FD or with other neurological conditions were excluded. The personal characteristics of each subject are detailed in Table 1. The hospital Helsinki committee specifically approved this study (approval number 0544-10-HMO). In addition, 16 healthy adults, without orthopaedic or neurologic imparments, recruited using a convinience sample, were tested at the gait laboratory. The two groups were matched by gender.

**Table 1:** Personal characteristics of the study participants (n = 12).

		S1	S2	S3	S4	S5	S6	S7	S8	S9	S10	S11	S12
Age [years]		16	43	25	23	19	19	10	35	24	20	45	24
Gender^¥^		F	M	F	M	M	F	M	F	M	F	M	M
Height [cm]		146	180	145	151	167	163	125	165	175	152	163	167
Weight [kg]		39	47	40	39	46	55	24	44	42	40	46	51
Body mass index [kg/m^2^]		18.3	14.5	19.0	17.1	16.5	20.7	15.4	16.2	13.7	17.3	17.3	18.3
Age that began walking (months)		24	24	18	12	18	10	32	18	24	14	NA	18
Walking aids		-	Walker	-	Walker	-	-	-	Walker	-	-	-	-
Ataxia^§^		+	+++	+++	++	+	++	++	+++	+++	+++	+++	++
Kyphoscoliosis^§^		++	++	+++	++	+	++	+	+	++	+++	+++	++
Spinal intervention		Yes	No	Yes	Yes	No	No	No	Yes	No	No	No	Yes
History of fracture (location)		Foot (twice)	Patella	Arm, ankle	-	Nose	-	-	-	Arm	-	Hand	Hand
Bone density of hip neck (z-score)		-2.6	-3.8	-1.6	-2.2	-2.4	-1.7	-2.5	-1.1	-3.2	-3.0	-2.4	-3.0
Number of falls in a month		0.5	1	2	2	0.5	1	1	1	4	1	4	0
Deep foot vibration threshold [V]	R	20	11	12	12	30	14	NA	26	28	14	30	30
	L	22	12	10	10	31	16	NA	28	26	15	32	32
Femoral anteversion [°]	R	32	17	NA	NA	8	-8*	NA	NA	25	NA	NA	NA
	L	19	16	NA	NA	0	-3*	NA	NA	28	NA	NA	NA
External tibial Torsion [°]	R	34	51	NA	NA	28	45	NA	NA	39	NA	NA	NA
	L	29	35	NA	NA	21	23	NA	NA	34	NA	NA	NA
Limb external rotation [°]	R	2	34	NA	NA	38	53	NA	NA	14	NA	NA	NA
	L	10	19	NA	NA	28	26	NA	NA	6	NA	NA	NA

^¥^M = Male; F = Female

*Negative values of femoral anteversion indicate femoral retroversion

^§^Ataxia, lumbar lordosis and thoracic kyphosis are categorized as mild (+), moderate (++), severe (+++).

NA = Not Available.

### 2.2 Study tools and protocol

Each subject thoroughly read and signed an informed consent form pretrial. According to the Helsinky comittee approval for the FD group, personal data and clinical measures were retrieved from the clinical records, including X-rays or other imaging data of each subject. In a personal questionnaire prepared for this research, the subjects filled out their age, gender, weight, height and details of walking aids at the time of recruitment. Scans of subjects who performed lower limb Computed Tomography (CT) in the last year were collected.

Foot deep vibration sensation was assessed using a Quantitative Vibration threshold test (Biothesiometer by Bio-Medical Instrument Co. Newbury, Ohio). The biothesiometer was found to be a more accurate gauge of vibration threshold compared with a tuning fork, which is the prevalent method used by neurologists to assess vibration sensation [22]. This measure was found to be abnormal in most patients with FD and its abnormality is hypothesized to result from disturbed conduction of vibratory impulse trains, thereby reflecting the degree of progressiveness of the disorder [23]. The device was set perpendicularly to the skin of the toe of the subject without applying pressure. The stimulus amplitude was increased until the subject reported that he or she perceived vibration. A linear scale of arbitrary units, ranged 0 to 50, shows the applied voltage proportional to the square root of the amplitude of vibration.

Computational gait analysis was performed while subjects walked on a 5.5m-long walking path. First, video-recorded observational analysis through the coronal and sagittal planes was performed, in order to visually register the overall walking pattern and compensation mechanisms. Then, 19 passive reflective markers were taped to the pelvis and lower extremities of each subject for a static standing trial. The markers were placed according to the Vicon's Plug-in-gait model [24]. The four markers placed on the medial aspects of the femoral epicondyles and the tibia apexes of medial malleoli were removed for the gait trial. The authors of a systematic review regarding the reliability of 3D kinematic gait measurements concluded that clinically-acceptable errors are possible in gait analysis [25].

Additionally, 8 telemetric surface EMG electrodes (Trigno^™^ Wireless EMG, Delsys, Boston, MA, USA) were placed on each subject. As stated by the authors reviewing multichannel surface EMG in clinical gait analysis, the recording of EMG in subjects with abnormal gait patterns represents a unique opportunity to analyze locomotor commands issued to specific muscles responsible for progression and dynamic stabilization of the body segments [26]. The skin was prepared by shaving and cleaning the area with alcohol pads. The electrodes were attached to the skin parallel to the direction to the fibers of 4 muscles bilaterally, i.e. four on each leg: Medial Gastrocnemius, Tibialis Anterior, Semitendinosus and Rectus Femoris, at locations detected according to the recommendations of Surface Electromyography for the Non-Invasive Assessment of Muscles (SENIAM)[27]. The subjects were instructed to walk several times at a comfortable speed on the path. Dynamic EMG data were recorded at a frequency of 1500Hz. The gait of the subject was captured using a 4-camera system (Basler Scout, Basler AG, Germany) at a frequency of 120Hz. A mobile ceiling-mounted safety harness was applied when needed, e.g. when a subject had difficulties maintaining balance during gait or while turning around when approaching a wall.

### 2.3 Post analysis

The 3D coordinates of the passive markers were calculated using a commercial software (Simi Motion, Germany; [28]). Initial contact and toe-off were marked manually using the video footage and were exported along with the markers' 3D coordinates to a custom code created in LabView (version 13, National Instruments, Austin, Texas, USA), where the spatio-temporal parameters were automatically calculated. The calculated spatio-temporal parameters comprised of the gait velocity (in meters/sec), which is a quotient of the sum of several steps lengths (in meters) and the time (in seconds), and cadence (step rate calculated as number of steps in a minute), the spatial gait parameters, e.g. step length and base width, as well as temporal gait parameters, e.g. stance, swing, and double support duration. Temporal data are presented in seconds and also normalized to the gait cycle duration of the leg and presented as a percent of gait cycle. In addition to the conventional spatio-temporal data of gait, our code automatically calculates a byproduct of the spatio-temporal data, which is the Symmetry Index (SI) for the stance, swing, and double support durations, as well as a SI for the step length and base width. The SI is calculated according to the following equation [29]:

$$SI[\%]=\frac{\left|X_{L}-X_{R}\right|}{\frac{1}{2}\cdot (X_{L}+X_{R})}\cdot 100$$ (1)

where *X*_*L*_ and *X*_*R*_ are the values of a spatial or temporal parameter of the left and right leg, respectively. The SI ranges between '0' for complete symmetry and '200' for asymmetry. The SI is the method most commonly used and cited in publications on gait symmetry [30]. The average and the SD of the spatiotemporal and SI were calculated from the measurement results of the recorded gait cycles of each subject.

The gait kinematics were calculated using a commercial research software for 3D motion capture data analysis and modeling (Visual 3D, C-Motion, MD, USA), found to be reliable across multiple laboratories [31].

A self-designed LabView code was used to read the EMG raw data, and perform baseline correction, filtering (band-pass filter of 10-500Hz), and full signal rectification. Then, filtered and rectified EMG data were Root Mean Squared (RMS) for consecutive segments of 50ms. The activation time of each muscle was marked where the EMG signal exceeded 18% of the peak RMS value [26,32]. This was compatible with the visual inspection of the data by a trained physician (IS) for the onset and offset muscle activation timings. The overall activation time of each muscle was normalized by the time of the gait cycle. For each muscle, the total activation time as a percentage of the stance and swing phase was calculated, as well as the onset time in each of the two gait stages.

Statistical analyses were performed using the SPSS (Version 24, IBM SPSS statistics). Due to the small sample size, descriptive statistics are presented as median and interquartile range (IQR), and non-parametric statistics were utilized. The Mann-Whitney Signed-Ranks test was used to compare between the entire FD group and healthy group.

Spearman correlations were performed to correlate the quantitative data of spatio-temporal gait parameters, gait symmetry and EMG parameters of the individuals with FD. Ataxia was classified by a trained physician observing the videos recorded through the coronal and sagittal planes. Several steps of gait were viewed, and the level of ataxic gait was categorized as either mild, moderate or severe according to the base width and variability of the base width and step length. This classification method is routinely performed at our gait laboratory and is done by videos alone, before quantitative data are available to the physician), crouch gait (yes/no, using the quantitative results of the sagittal kinematic data so that crouch gait was defined as a minimum knee flexion angle greater than 10°; [33]), and the number of reported falls in the last month (less than 1, 1, or above 1 fall a month). Results were considered statistically significant if p<.05.

## 3. Results

Twelve participants with FD (7 males and 5 females; mean±SD age 25.3±10.6) and 16 healthy participants (6 males and 10 females, mean±SD of age 35.9±11.9 years old, and height 1.70±0.05m), participated in the study. The description of each subjects with FD is presented in Table 1. Nine out of 12 participants with FD were able to ambulate without a walker. The FD subjects began walking at an average age of 19.3 months, ranging 10 to 32 months.

The average Body Mass Index (BMI) of the 11 adult FD subjects, was 17.2 kg/m^2^ (2 classified in the normal range, and 9 were underweight so that 5 were classified with “mild thinness”, 2 with “moderate thinness”, and 2 with “severe thinness” according to the WHO) and a normal BMI of 15.4 kg/m^2^ for the 10 year old subject. All of the FD subjects had various degrees of kyphoscoliosis and 5 out of 12 subjects underwent spinal fusion. The frequency of fractures reported in our study was as follows: 5 out of 12 FD subjects reported one facture, and 2 out of 12 FD subjects reported 2 fractures. Four of the five subjects with T-score below -2.5 at the femoral neck, which is a cutoff for osteoporosis according to the WHO, reported fractures. Also, 6 subjects out of 11 had abnormal deep foot vibration thresholds bilaterally, i.e. above 10V [34]. The level of ataxic gait was mostly severe (6 out of 12), then moderate (4 out of 12) and low (2 out of 12), whereas the control group showed no evidence of ataxic gait. Also, 3 out of 12 subjects demonstrated crouch gait.

The spatio-temporal gait parameters and gait symmetry of each FD subject are pressented in Table 2. For completion of the reported data, the dynamic EMG results of each subject are presented in the Appendix (S1 Table). Also, the gait kinematics of each subject are presented as an Appendix for the sagittal plane (S1 Fig). We did not include kinemaitcs in the frontal and transversal planes since we suspected a possible misalignment of the medial-lateral axis of the femur coordinate system, defined by markers placed on the medial and lateral epicondyles. This misalignment might have resulted in cross talk between the sagittal and coronal planes, and caused large varus movement calculation during the swing phase [35].

**Table 2:** Spatio-temporal gait data and symmetry indices of the subjects. In the right coulombs are the median (Med) value and inter-quartile range (IQR).

														FD patients		Controls	
		S1	S2	S3	S4	S5	S6	S7*	S8	S9	S10	S11	S12	Med	IQR	Med	IQR
Velocity [m/s]		0.21	0.2	0.92	0.78	1.09	0.89	0.69	0.55	0.67	0.51	0.88	0.65	0.7	0.4	1.1	0.3
Cadence [steps/min]		129.7	33.7	118.6	107.3	116.4	84.5	118.8	118.8	86.9	87.9	144.6	101.5	107.3	31.3	106.5	11.8
Temporal data [s]	Step time	0.47	1.76	0.51	0.56	0.52	0.71	0.51	0.94	0.70	0.69	0.53	0.6	0.60	0.18	0.56	0.06
	Stance duration	0.56	2.73	0.65	0.76	0.63	0.87	0.69	1.30	0.80	0.84	0.69	0.8	0.80	0.19	0.68	0.09
	Swing duration	0.37	0.82	0.38	0.39	0.40	0.54	0.34	0.62	0.57	0.45	0.41	0.4	0.41	0.16	0.43	0.03
	Double support	0.10	0.96	0.14	0.18	0.12	0.16	0.16	0.36	0.10	0.20	0.12	0.2	0.16	0.08	0.13	0.04
	Single support	0.37	0.82	0.38	0.39	0.40	0.54	0.34	0.62	0.55	0.45	0.41	0.4	0.41	0.15	0.43	0.04
Temporal data [%GC]	Stance duration	60.4	76.9	63.4	66.3	61.4	61.8	67.3	67.6	58.3	65.1	62.9	64.3	63.9	4.9	61.3	2.2
	Swing duration	39.6	23.2	36.6	33.7	38.6	38.2	32.7	32.4	41.7	34.9	37.1	35.8	36.2	4.9	38.7	2.2
	Double support	10.8	28.5	13.1	16.6	11.3	10.9	17.6	18.5	9.1	13.8	12.3	13.5	13.3	5.7	11.4	2.6
	Single support	38.8	22.8	36.1	35.2	38.5	39.0	34.2	32.0	40.3	33.1	41.7	35.7	35.9	4.9	38.3	2.9
Spatial data [cm]	Stride length	18.9	69.8	93.7	84.4	112.9	128.4	70.5	104.7	93.3	66.9	98.2	93.3	93.3	24.4	121.5	14.3
	Step length	9.5	35.1	46.6	43.5	56.1	63.6	34.6	51.4	46.4	35.1	46.2	46.2	46.2	9.7	60.5	7.0
	Base width	43.5	19.0	17.5	15.7	14.4	7.3	15.0	25.8	20.3	17.6	11.7	17.5	17.5	4.6	9.9	4.1
Foot progression [°]		25.6	5.9	14.8	19.3	12.2	-0.9	3.1	3.1	6.0	8.0	29.9	15.1	12.2	11.3	6.3	4.5
Symmetry index (SI)	Step time	1.7	16.9	0.5	13.5	15.3	11.3	6.5	1.8	18.9	18.9	10.4	10.9	11.1	10.4	3.9	4.5
	Stance duration	6.0	0.3	6.3	6.0	8.5	11.2	6.6	8.7	13.1	23.8	1.3	7.6	7.1	3.3	2.5	2.2
	Swing duration	9.1	1.9	12.2	3.4	14.6	9.6	8.6	35.5	13.7	12.1	27.6	12.2	12.2	5.0	3.0	4.3
	Double support	32.8	32.1	23.6	45.6	17.7	8.0	15.4	31.7	36.9	1.8	27.2	25.4	26.3	15.2	10.5	14.8
	Step length	83.3	31.9	5.3	2.8	1.5	16.0	2.0	24.8	7.3	14.4	4.6	8.9	8.1	14.1	4.3	6.2
	Base width	8.9	37.2	17.7	55.3	4.0	14.3	4.5	22.6	31.9	1.1	69.2	16.0	16.9	25.4	13.6	26.6

*Since S7 is 10 years old, data of velocity, cadence, temporal data in seconds, spatial data and foot progression of this subject, were not included in the calculation of the median and IQR.

GC = Gait Cycle

Statistical analysis showed that compared to healthy subjects, FD subjects ambulated with increased gait asymmetry in parameters of step time (z = -2.322, p = .020), stance and swing durations (z = -2.966. p = .003), and double support duration (z = -2.832, p = .005). Although there were no significant differences in their cadence, compared to the healthy subjects, the gait velocity of the FD subjects was significantly lower (z = -3.971, p<.001). The percentage of stance duration in relation to the gait cycle was significantly higher, while the swing duration was significantly lower in FD subjects compared to healthy subjects (p = .015). Also, their step length was significantly shorter (p<.001) and their base width was significantly wider (p = .009). As described in the Introduction, an increase base width is a previously-identified symptom of ataxic gait. Further analysis showed that in the FD group, the youger subjects (age 20 years and below; 5 out of 12) had significantly better symmetry of their base width (median 4.5) compared to the older subjects (median 31.9; p = 0.012). We also found that the level of ataxic gait strongly correlated with age (r = .907, p<.001).

One of the prominant findings from the kinematic data are the hip and knee transversal rotations recorded during gait, either internal or external, seperately in each leg. However, as mentioned above, this should be further explored and verified using methods that reduce cross talk between the sagittal and coronal planes. Five subjects had lower limb CT and the osseous torsions of the femur and tibia were measured by a trained radiologist (NH). All five subjects with CT showed unormal femoral and/or tibial osseous rotations.

**Fig 1:**
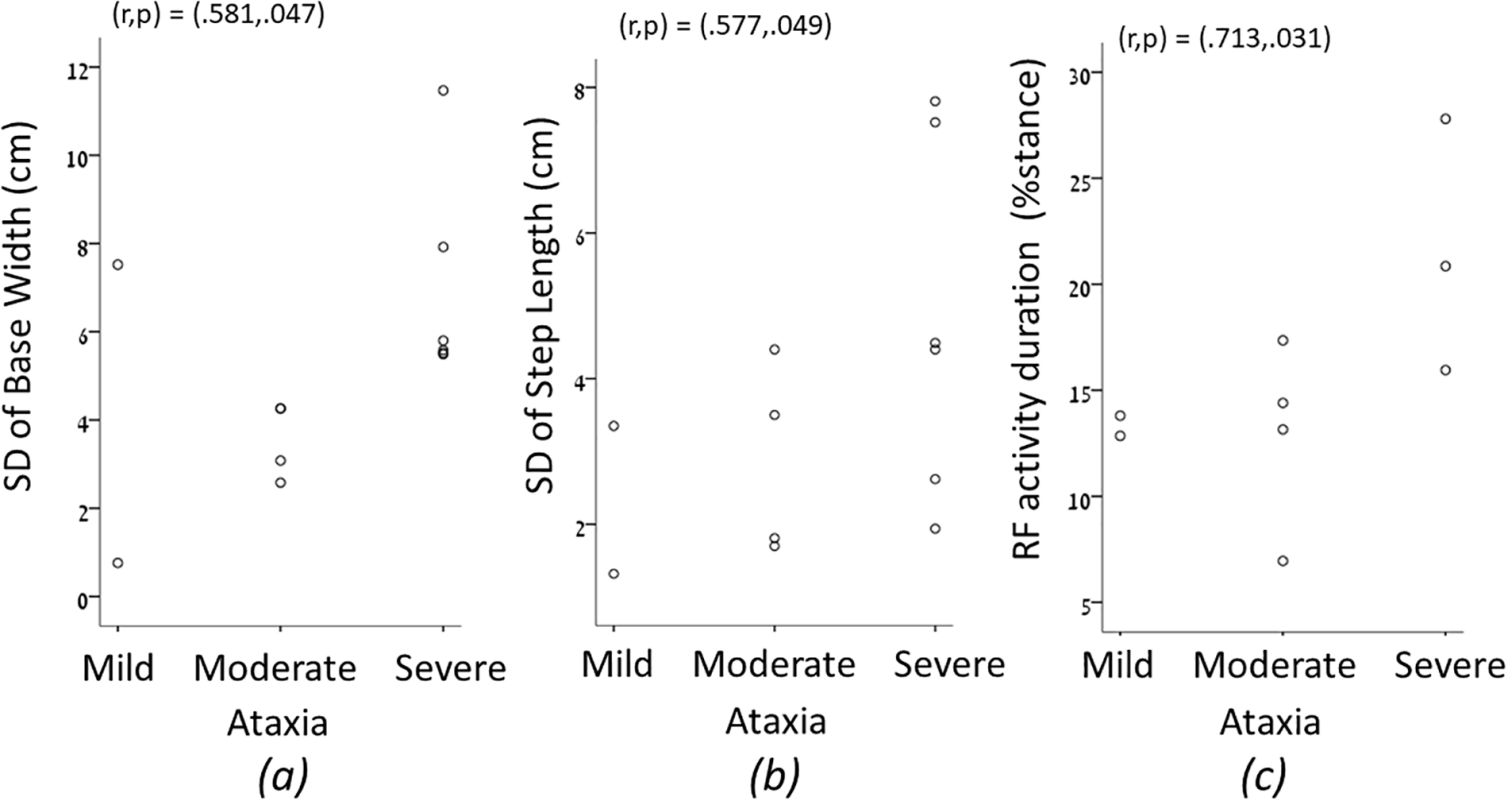
A scatter plots derived following correlations between the level of ataxia and the Standard Deviation (SD) of the base width and step length, as well as the activity duration of the rectus femoris (RF) during the stance phase.

**Fig 2:**
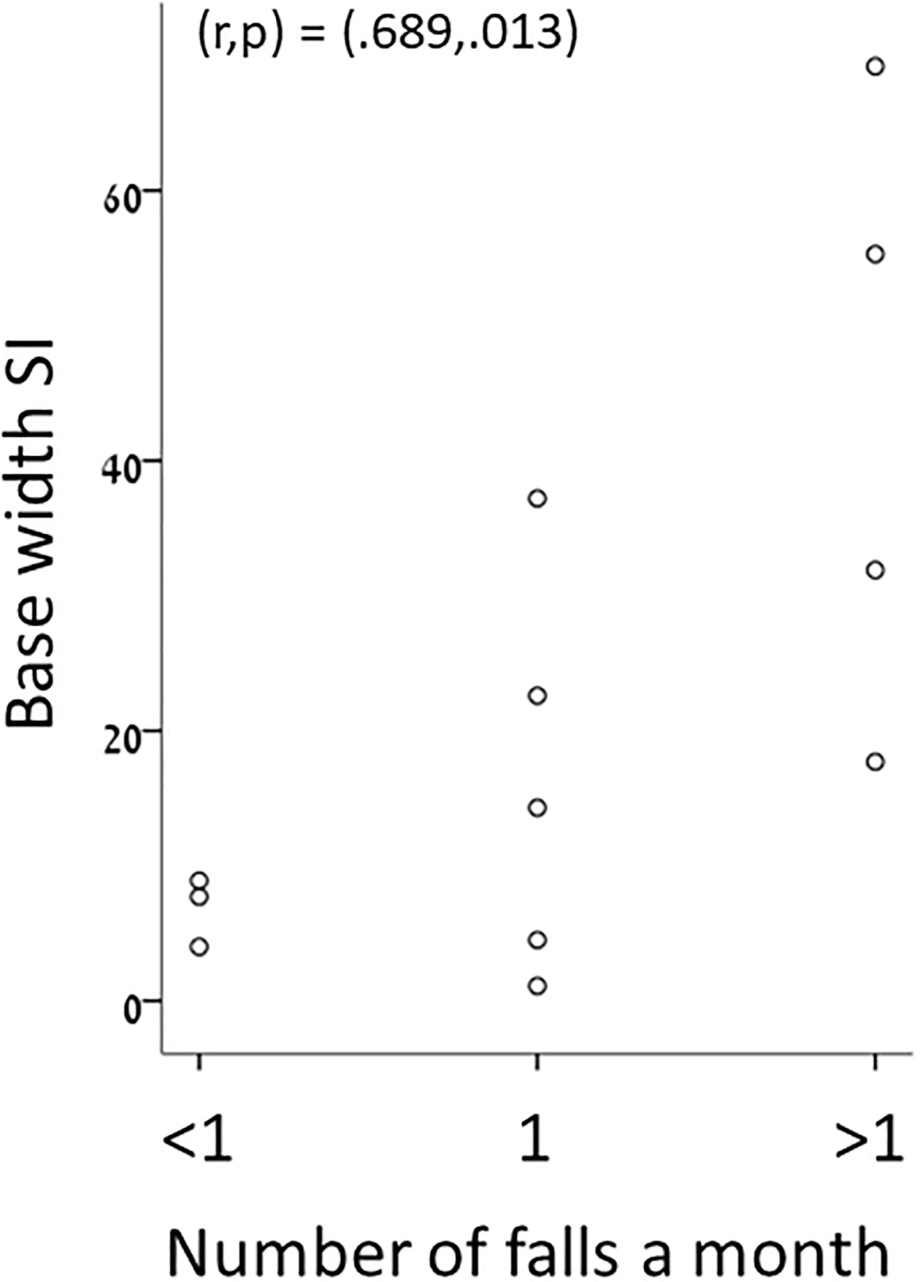
A scatter plot showing the correlation between the number of falls and the Symmetry Index (SI) of the base width.

Scatter plots derived following correlations between the level of ataxia and the SD of the base width and step length, as well as the activity duration of the rectus femoris during the stance phase are depicted in Fig 1. These graphs depict the increase in SD of spatial gait parameters and in activation of the knee extensor as the severity of ataxia increases. A scatter plot showing the correlation between the number of falls and the SI of the base width is depicted in Fig 2, showing correlation between an increase in the number of falls and increase in gait asymmetry. The number of falls did not correlate with the transversal rotations during midstance.

## 4. Discussion

In this study we describe the medical condition of individuals with FD, compare their gait characteristics to healthy individuals, and correlate between gait measures, presence of unstable gait pattern and frequency of falls. This is the first study to report the results of a comprehensive gait analysis in the FD population. All subjects with FD presented a clinical ataxic gait pattern. The number of falls correlated with base width asymmetry.

An unexplored condition that may contribute to the unbalanced gait is the osseous abnormalities in this population. In this study, the degree of tibial and/or femoral osseous anteverssion could not be correlated with the degree of transversal rotation during gait due to the small sample size. Previous studies of gait characteristics in children with CP showed that femoral anteversion and tibial torsion explain only 25% of variance in regression analysis of foot progression angle [36] and that children with tibial torsion had delayed peak knee flexion and high hip rotation during gait [37]. Some of the following abnormalities were reported in 136 individuals, aged 3 months to 64 years with FD based on a physical examination and radiographs [15]: Spine deformity occurred in 86% of the subjects by the age of 15 years old, 53% of whom had scoliosis, 44% had kyphoscoliosis and 4% had kyphosis without scoliosis, and foot deformities were documented in 11.7%. As explained by Bar-On et al [15], although the deformities may appear to be of neurogenic origin, the neurological deficit associated with FD, affects afferent pathways. However, the agonist-antagonist muscle balance is normal and without spasticity. Therefore, the cause for increased prevalence of leg deformities in individuals with FD remains unclear. In the aforementioned rare study of a large sample size of individuals with FD [15], delayed walking was noted in 72% of the 130 patients who were of walking age. All 64 individuals, re-examined after a mean duration of follow-up of sixty-five months, had ataxic gait, although with high variability of its degree [15]. Surprisingly, none of the re-examined patients used an assistive device for gait.

In this study, only 4 out of 12 subjects did not attain walking milestone by the age of 18 months (which is the classification for delayed walking by the World Health Organization) [38]. The low BMI found in most of the FD subjects in this study, is similar to the BMI values of FD patients reported by Maayan et al [39]. Low BMI may suggest muscle atrophy and weakness, endangering the mobility of the patients [40].

Compared to healthy subjects, the FD subjects who participated in the current study ambulated slowly, with significantly increased gait asymmetry of several temporal parameters. Furthermore, their unstable gait was characterized by significantly prolonged stance duration, reduced step length, and wider base width, compared to healthy subjects. As was shown in previous literature, also in our study, 9 out of 12 subjects had moderate to severe ataxia. The cause of ataxic gait in this population has a few possible hypotheses. The latest hypothesis includes two main factors: cerebellar atrophy and a certain level of deep sensation impairment. The latter, decreased vibration sensation, was found in 6 of our FD subjects. Unsurprisingly, the clinical ataxic gait levels were correlated with the SD of the step length and base width, reflecting high inconsistency of step length and base width during ataxic gait. However, we expected all subjects to walk with wider step width, but this was not conclusive in our study as only four had increased base width.

Another interesting finding of this study is that 3 out of 12 subjects ambulated with crouch gait. Crouch gait has been shown to increase energy consumption[19] so that fatiguing activities are abandoned and participation in daily life activities may be reduced. Crouch gait is frequent in patients with CP, but has been shown in other pathologies, e.g. Dravet syndrome [41]. It is caused either by weakness of ankle plantar flexors or the knee extensors, spasticity or contracture of hip and knee flexors, lever arm dysfunction (disruption of muscle-generated moment due to shortened moment arm), or combinations of these [17,42]. The FD subjects may compensate for balance deficiencies by lowering their center of mass. Furthermore, the rotational profiles of lower extremity joints reveal increasing lever arm dysfunction, which may induce crouch gait [43]. The presence of crouch gait in the subjects reported herein was probably not related to muscle weakness or tendon tightness since there were no such findings following our physical exams.

A moderate correlation was found between the SD of the base width and step length and ataxia levels (Fig 1a and 1b). The relationship between step length and gait cadence, termed Walk Ratio (WR), has been shown to decrease in populations with Multiple Sclerosis [44], Parkinson’s disease [45], and following stroke [46], compared with controls. This measure reflects the quality of gait control [44]. In our study, 3 out of 12 subjects had WR that was below the normal range, 3 out of 12 subjects had normal WR, and 4 out of 12 subjects had WR that was above the normal range. Accordingly, this measure should also be further explored in this population as a predictor of falls.

Falls prevention is a great concern for individuals with FD and their families. Previous studies identified several gait parameters as strong predictors of falls. In a 1-year study of 96 elderly women (49% fallers), double support duration (% of the gait cycle) and the symmetry of temporal gait parameters were strong predictors of falls. In 48 patients with symptoms of a cerebellar ataxia, the spatio-temporal gait variability in the fore-aft direction was associated with the history of falls [14]. On the contrary, the step length and gait velocity, were not good predictors of falls in 352 community-dwelling older persons (39% fallers) [47]. Our finding shows strong correlation between the SI of the base width and falls in individuals with FD. Increasing the base of support of patients with FD, e.g. administrating a walker, might increase the symmetry of the base width during gait and decrease the number of falls. However our results should be reproduced with a larger sample size to strengthen the basis for our conclusions. Additionally, perturbation treatment and dynamic balance exercises may be recommended in order to improve compensatory strategies.

In our study, we found no correlation between the transversal rotations during midstance and the number of falls or ataxic gait. This is the first study to investigate correlation between transversal joint rotations of the hip and knee during gait and frequency of falls. Although we investigated a small sample-sized group, our conclusion according to these preliminary data is that intervention for rotations should not be the first priority for treatment for gait improvement, as frequently performed for patients with CP since we surmise from our results that it is not the cause of falls. Further research is warranted in a larger sample size of individuals with FD.

Higher activity duration of the Rectus Femoris during the stance phase correlated with higher severity level of ataxic gait, but did not correlate with the number of falls. Since unstable gait requires enhanced muscle activity, particularly from large stabilizing muscles, this finding is logical. Prolonged activities of these large muscles can lead to fatigue, that may be prevented using walking aids.

The main limitation of this study is the small sample size. Considering that FD is a rare disease and adults with FD can rarely ambulate without human assistance, this sample is adequate but cannot be generalize to individuals who cannot ambulate. Another limitation is that the CT scans for quantifying tibial and femoral anteversion and EMG data were not available for all subjects. Furthermore, our chosen marker set poses a limitation since it does not allow modeling of the subtalar kinematics, which might differ between healthy individuals and individuals with FD.

In conclusion, this in-depth quantitative gait analysis of patients with FD has not been previously described in the literature and revealed ataxic gait. Future investigation of this population should include quantification of osseous rotations of the lower limb in order to fully understand its effect on their gait pattern and falls.

## Supporting information

S1 Fig The kinematic report for each subject. The pelvis, hip, knee and ankle are presented in rows from top to bottom, respectively for the sagittal plane. The right (red line) and left (blue line) side are presented and normlized to the gait cycle. The vertical lines in the graph represent the end of the stance phase of each leg. The grey area is the normal data collected at our lab.
(DOCX)

S1 Table Timings and durations of the EMG activities of the monitors muscle of each subject (n = 9). (DOCX)
