# Supplementary figures and images for: Characteristics of ataxic gait in familial dysautonomia patients
Source: PLoS One. 2018 Apr 26;13(4):e0196599. doi: 10.1371/journal.pone.0196599 (PMC5919612; doi:10.1371/journal.pone.0196599)

| 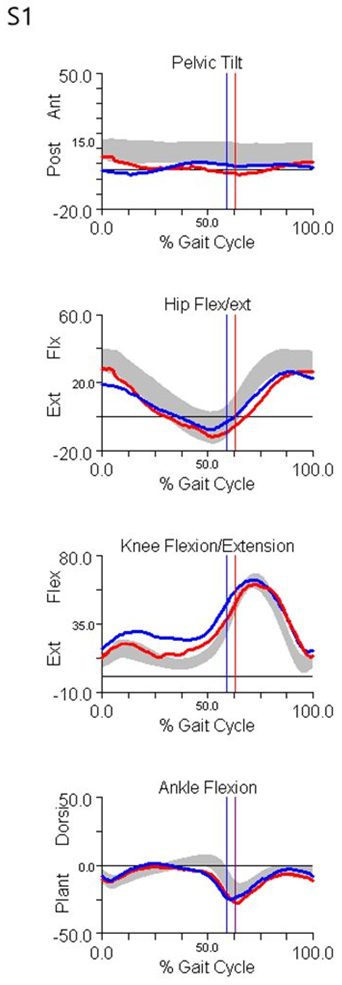 | 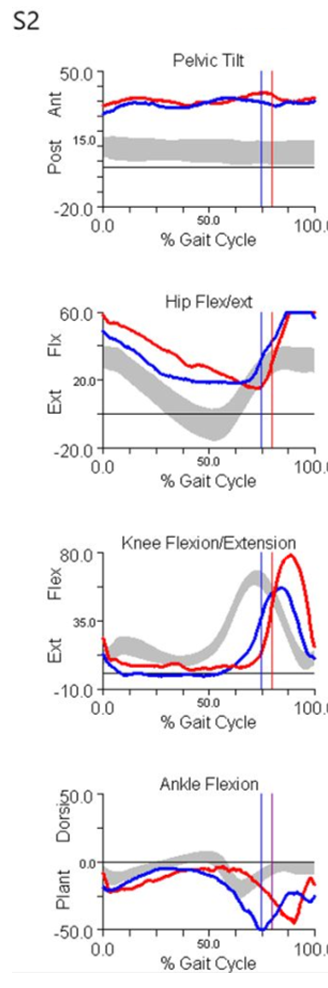 | 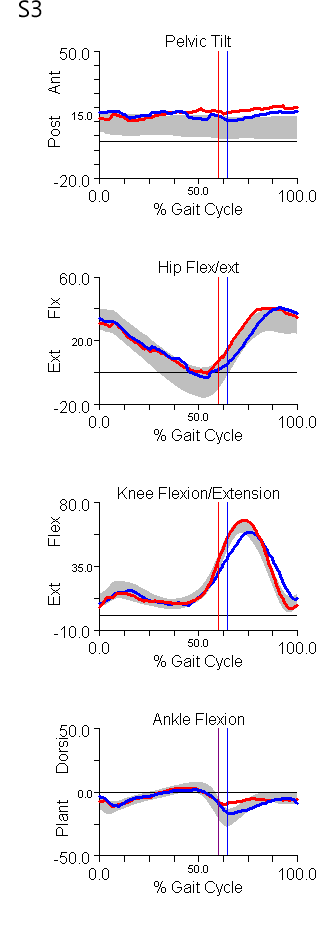 | 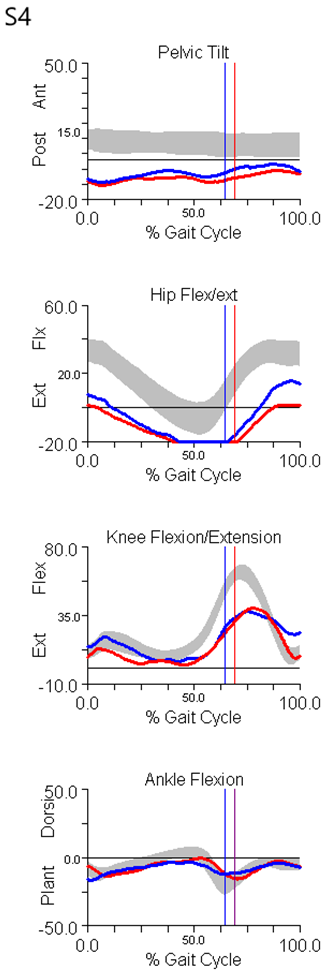 |
| --- | --- | --- | --- |
| 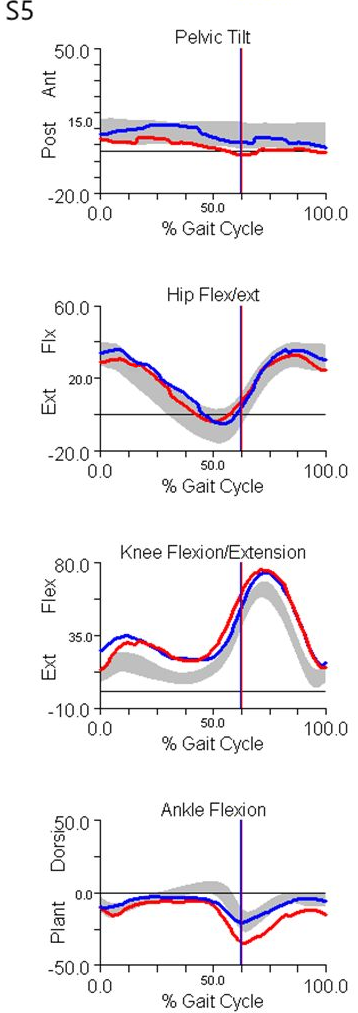 | 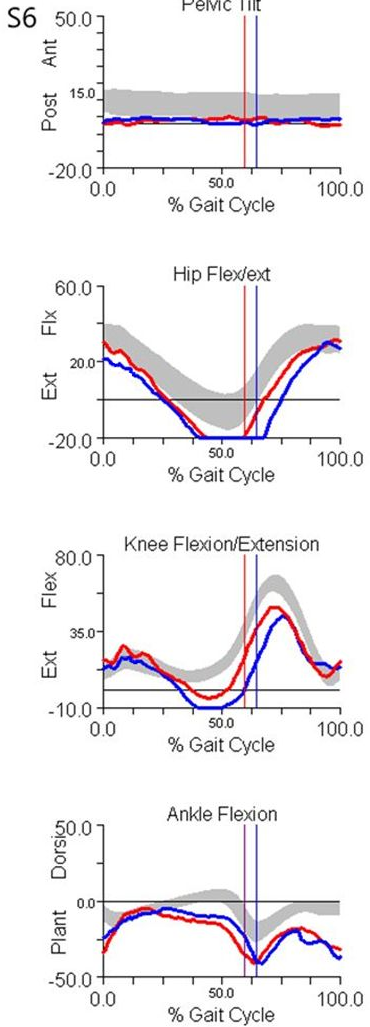 | 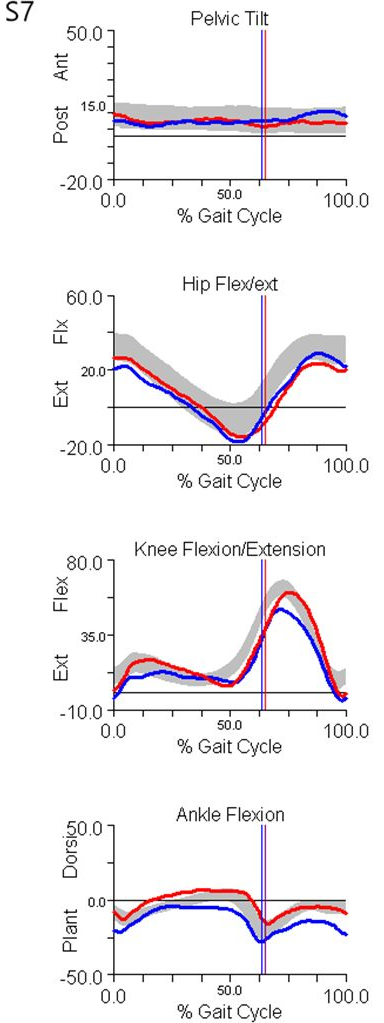 | 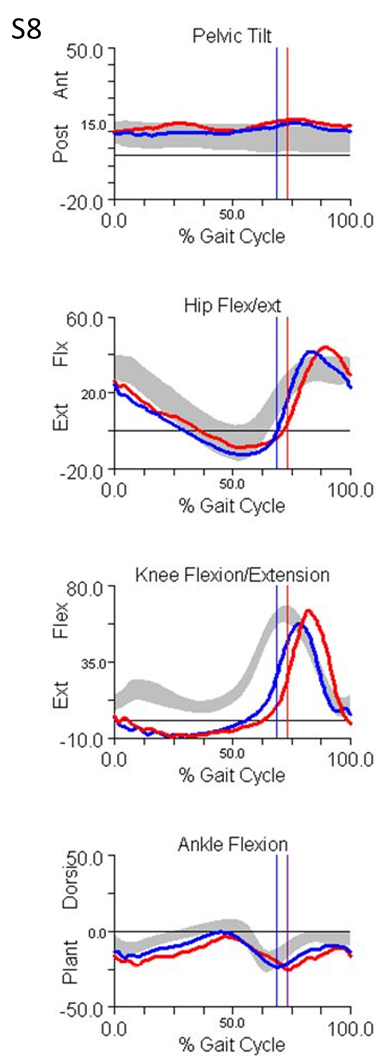 |

| 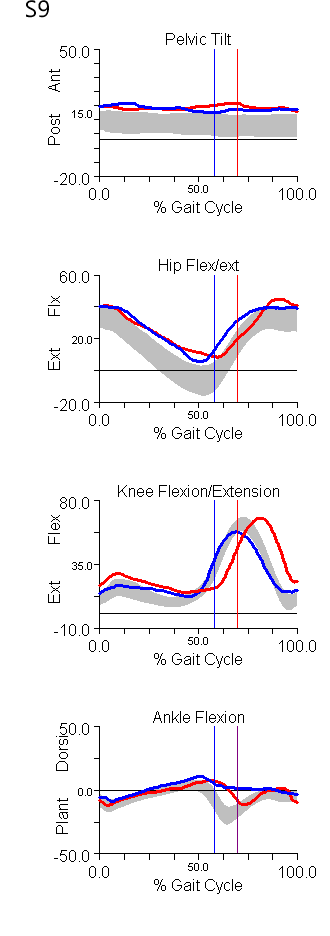 | 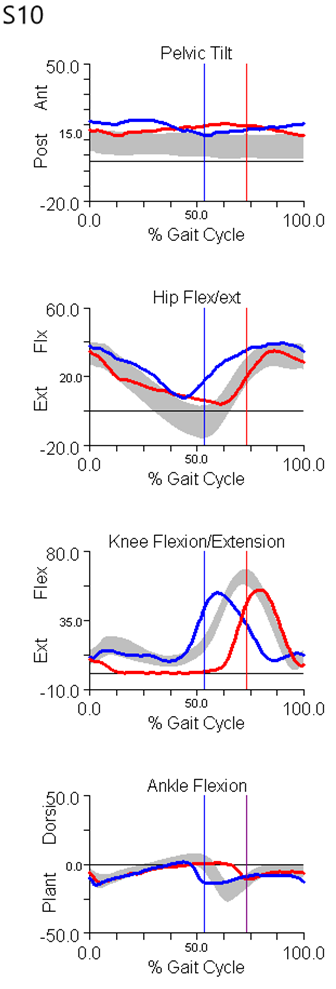 | 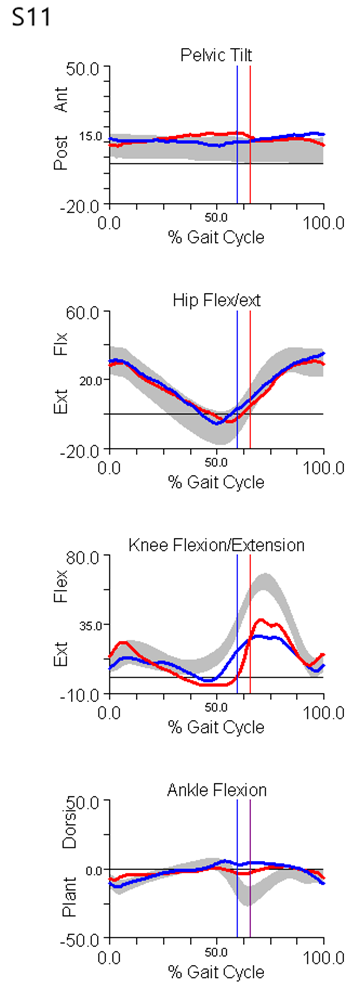 | 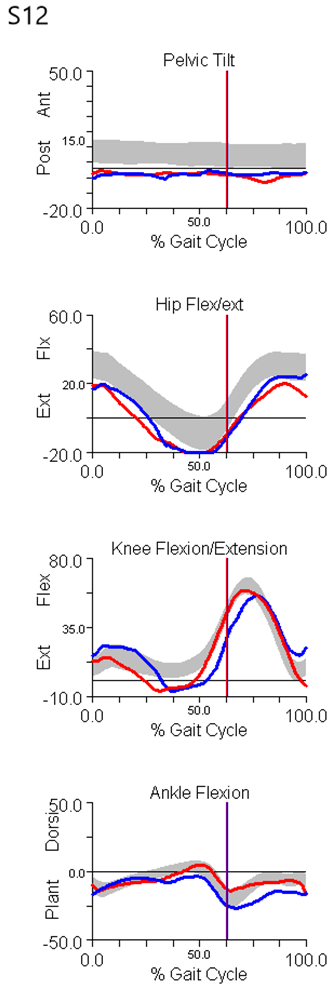 |
| --- | --- | --- | --- |

Supplement: S1 Fig — The pelvis, hip, knee and ankle are presented in rows from top to bottom, respectively for the sagittal plane. The right (red line) and left (blue line) side are presented and normlized to the gait cycle. The vertical lines in the graph represent the end of the stance phase of each leg. The grey area is the normal data collected at our lab. (DOCX) [file pone.0196599.s001.docx]
