# Supplementary material for: Characteristics of ataxic gait in familial dysautonomia patients
Source: PLoS One. 2018 Apr 26;13(4):e0196599. doi: 10.1371/journal.pone.0196599 (PMC5919612; doi:10.1371/journal.pone.0196599)
Supplement: S1 Table — (DOCX) [file pone.0196599.s002.docx]

S1 Table: Timings and durations of the EMG activities of the monitors muscle of each subject (n=9)

|  |  |  | **S1** | **S2^*^** | **S4** | **S5** | **S6** | **S7** | **S8** | **S11** | **S12^*^** | **Median** | **IQR** |
| --- | --- | --- | --- | --- | --- | --- | --- | --- | --- | --- | --- | --- | --- |
| Gastrocnemius | Activity time (%ST) | L | 27.4 | 18.3 | 18.1 | 20.3 | 13.7 | 16.8 | 21.5 | 8.7 | 4.7 | 18.1 | 6.6 |
|  |  | R | 19.6 | 25.3 | 28.8 | 16.3 | 32.3 | 22.7 | 7.0 | 8.4 | 11.0 | 19.6 | 14.3 |
|  | Activity time (%SW) | L | 1.0 | 6.0 | 0.6 | 13.9 | 6.9 | 2.5 | 7.0 | 0.6 | NA | 4.3 | 4.9 |
|  |  | R | 0.9 | 6.3 | 8.4 | NA | 8.5 | 3.4 | NA | NA | NA | 6.3 | 2.1 |
|  | Onset time (%ST) | L | 0.5 | 0.1 | 3.3 | 0.1 | 12.3 | 0.1 | 0.2 | 0.9 | 16.2 | 0.5 | 3.2 |
|  |  | R | 0 | 0.3 | 1.8 | 8.3 | 0 | 1.2 | 5.9 | 0.8 | 2.1 | 1.2 | 1.3 |
|  | Onset time (%SW) | L | 0.6 | 0 | 22.0 | 0 | 0.1 | 11.1 | 0.4 | 34.5 | NA | 0.5 | 13.8 |
|  |  | R | 0.3 | 0 | 0 | NA | 0 | 28.9 | NA | NA | NA | 0.0 | 0.3 |
| Tibialis Anterior | Activity time (%ST) | L | 3.4 | 14.7 | 15.4 | 3.7 | 18.4 | 22.8 | 19.9 | 5.2 | 9.1 | 14.7 | 9.3 |
|  |  | R | 7.6 | 21.2 | 11.9 | 6.7 | 7.6 | 22.4 | 8.7 | 8.0 | 9.3 | 8.7 | 3.9 |
|  | Activity time (%SW) | L | 6.8 | 9.7 | 6.1 | 2.1 | 11.4 | 4.1 | 12.4 | 14.3 | 0.5 | 6.8 | 7.3 |
|  |  | R | 14.5 | 10.9 | 10.4 | 10.3 | 7.6 | 11.9 | 8.1 | 1.2 | 0.8 | 10.3 | 3.3 |
|  | Onset time (%ST) | L | 0.3 | 0.3 | 0.8 | 0.2 | 2 | 0.5 | 0.1 | 0 | 1.7 | 0.3 | 0.6 |
|  |  | R | 0.4 | 0 | 0 | 0.1 | 35.9 | 0.1 | 1.2 | 0.5 | 16.0 | 0.4 | 1.1 |
|  | Onset time (%SW) | L | 5.4 | 0 | 0.9 | 0.5 | 0 | 0.3 | 0.6 | 0.6 | 0 | 0.5 | 0.6 |
|  |  | R | 12.7 | 0.6 | 0.3 | 0 | 11.5 | 0 | 0.2 | 4.8 | 2.2 | 0.6 | 4.6 |
| Rectus Femoris | Activity time (%ST) | L | 18.5 | 24.9 | 12.7 | 20.1 | 8.1 | 7.1 | 10.5 | 18.2 | 17.1 | 17.1 | 8.0 |
|  |  | R | 9.1 | 30.7 | 16.1 | 5.6 | 18.2 | 6.8 | 21.4 | 23.5 | 17.6 | 17.6 | 5.3 |
|  | Activity time (%SW) | L | 15.8 | 6.8 | 8.1 | NA | NA | NA | 7.7 | 3.4 | 1.2 | 7.3 | 3.8 |
|  |  | R | NA | 3.9 | 8.8 | 1.5 | NA | NA | 11.1 | 6.6 | 6.4 | 6.5 | 3.1 |
|  | Onset time (%ST) | L | 0 | 0 | 0 | 0 | 0.2 | 0 | 0.1 | 0.5 | 0 | 0.0 | 0.1 |
|  |  | R | 2.4 | 0.1 | 0 | 1.2 | 0.7 | 5.3 | 0 | 0 | 2.9 | 0.7 | 2.4 |
|  | Onset time (%SW) | L | 1.6 | 1.5 | 5.7 | NA | NA | NA | 9.6 | 30.1 | 35.8 | 7.7 | 18.8 |
|  |  | R | NA | 0 | 6.6 | 38.7 | NA | NA | 0 | 5.8 | 3.4 | 4.6 | 4.7 |
| Semitendinosus | Activity time (%ST) | L | 26.4 | - | 12.5 | 16.7 | 20.8 | 26.0 | 18.4 | 19.6 | - | 19.6 | 5.9 |
|  |  | R | 22.8 | - | 16.9 | 24.0 | 18.5 | 14.1 | 0.8 | 15.4 | - | 16.9 | 5.9 |
|  | Activity time (%SW) | L | 5.1 | - | 2.5 | 9.1 | 9.9 | 7.2 | 2.4 | 6.1 | - | 6.1 | 3.9 |
|  |  | R | 4.5 | - | 5.4 | 3.6 | 17.5 | 3.9 | 3.6 | 5.1 | - | 4.5 | 1.5 |
|  | Onset time (%ST) | L | 0 | - | 0.4 | 5.6 | 0.5 | 1.6 | 0 | 0.1 | - | 0.4 | 0.8 |
|  |  | R | 0 | - | 1.9 | 0 | 0 | 0.9 | 9.0 | 1.2 | - | 0.9 | 1.1 |
|  | Onset time (%SW) | L | 1.4 | - | 21.9 | 0 | 0.8 | 0.2 | 0.2 | 18.1 | - | 0.8 | 9.6 |
|  |  | R | 0 | - | 24.5 | 20.2 | 1.9 | 27.5 | 0.6 | 2.3 | - | 2.3 | 20.3 |

NA = No Activity

^*^Data from the Semitendinosus are not presented due to technical complications encountered while acquiring the data.
